# Supplementary material for: Enhancing Wastewater Depollution: Sustainable Biosorption Using Chemically Modified Chitosan Derivatives for Efficient Removal of Heavy Metals and Dyes
Source: Materials (Basel). 2024 Jun 3;17(11):2724. doi: 10.3390/ma17112724 (PMC11173971; doi:10.3390/ma17112724)
Supplement: Supplementary file 1 [file materials-17-02724-s001.zip › materials-2923105-supplementary.pdf]

Enhancing Wastewater Depollution: Sustainable Biosorption Using Chemically Modified Chitosan Derivatives for Efficient Removal of Heavy Metals and Dyes

Jana Ayach [1,2], Luminita Duma [2,*], Adnan Badran [3], Akram Hijazi [1], Agathe Martinez [2], Mikhael Bechelany [4,5,*], Elias Baydoun [6] and Hussein Hamad [1]

1 Research Platform for Environmental Science (PRASE), Doctoral School of Science and Technology, Lebanese University, Beirut P.O. Box 657314, Lebanon; jana.ayach.1@st.ul.edu.lb (J.A.); akram.hijazi@ul.edu.lb (A.H.); hussein.hamad@ul.edu.lb (H.H.)
2 CNRS, ICMR UMR 7312, University of Reims Champagne-Ardenne, 51687 Reims, France; agathe.martinez@univ-reims.fr
3 Department of Nutrition, University of Petra, Amman P.O Box 961343, Jordan; abadran@uop.edu.jo
4 Institut Européen des Membranes (IEM), UMR-5635, University of Montpellier, Centre National de la Recherche Scientifique (CNRS), École Nationale Supérieure de Chimie de Montpellier (ENSCM), Place Eugène Bataillon, 34095 Montpellier, France
5 Functional Materials Group, Gulf University for Science and Technology (GUST), Mubarak Al-Abdullah 32093, Kuwait
6 Department of Biology, American University of Beirut, Beirut P.O. Box 110236, Lebanon; eliasbay@aub.edu.lb
* Correspondence: luminita.duma@univ-reims.fr (L.D.); mikhael.bechelany@umontpellier.fr (M.B.)

Section S1

Adsorbent characterization. The prepared chitosan powder and beads adsorbents were characterized using several techniques. The particle size analysis was conducted using Granulometric LA-250V2, particle size distributions based on the Fraunhofer theory. The SEM images were taken using a Hitachi S4800 machine after sputtering with a Polaron SC7620 Mini Sputter Coater. The BET analysis was conducted on a Teller V4.04H-1098 method. The XPS was conducted using an Escalab 250 apparatus, and by Jasco model V570 UV–Visible spectrophotometry. The FTIR was performed on the JASCO FTIR-6300 spectrometer (400-4000 cm-1). The zeta potential was measured using Zeta-Meter 4.0-4012. NMR spectra in solution were recorded on a Bruker Avance AVIII-600 NMR spectrometer using the Bruker TOPSPIN Software (Wisembourg, Germany), solid NMR (VNMRS 300MHz-VARIAN) were access to the majority of active nuclei in NMR. The XRD measurements were done with a PANAlytical Xpert-PRO diffractometer.

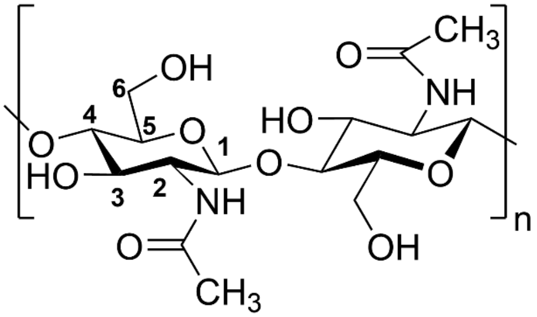

1: chitin

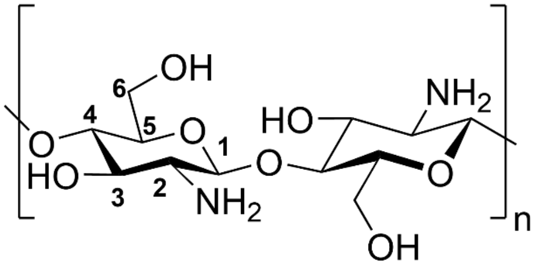

2: chitosan

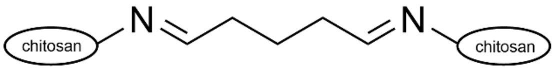

3: chitosan-based hydrogel

Figure S1 : Drawing of **1** chitin, **2** chitosan and **3** chitosan-based hydrogel structure.

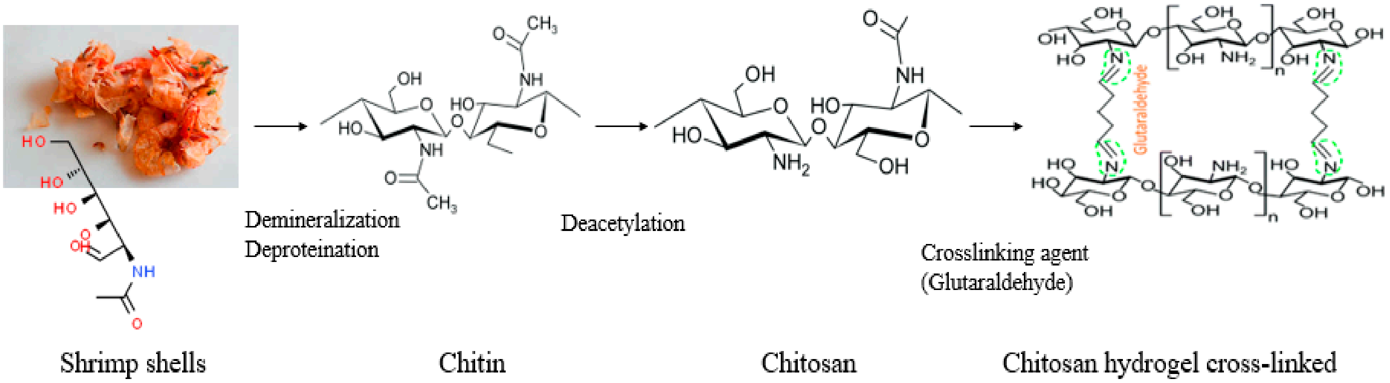

Figure S2. Brief scheme of chitosan hydrogel synthesis.

Results and discussion section:

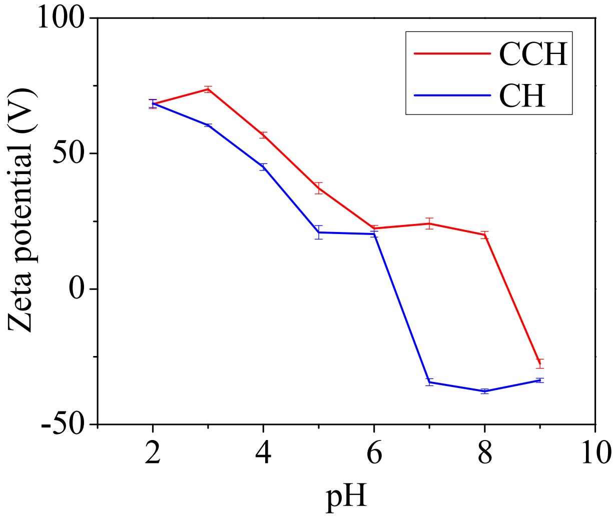

Figure S3. Zeta potential of commercial and extracted chitosan at different pH.

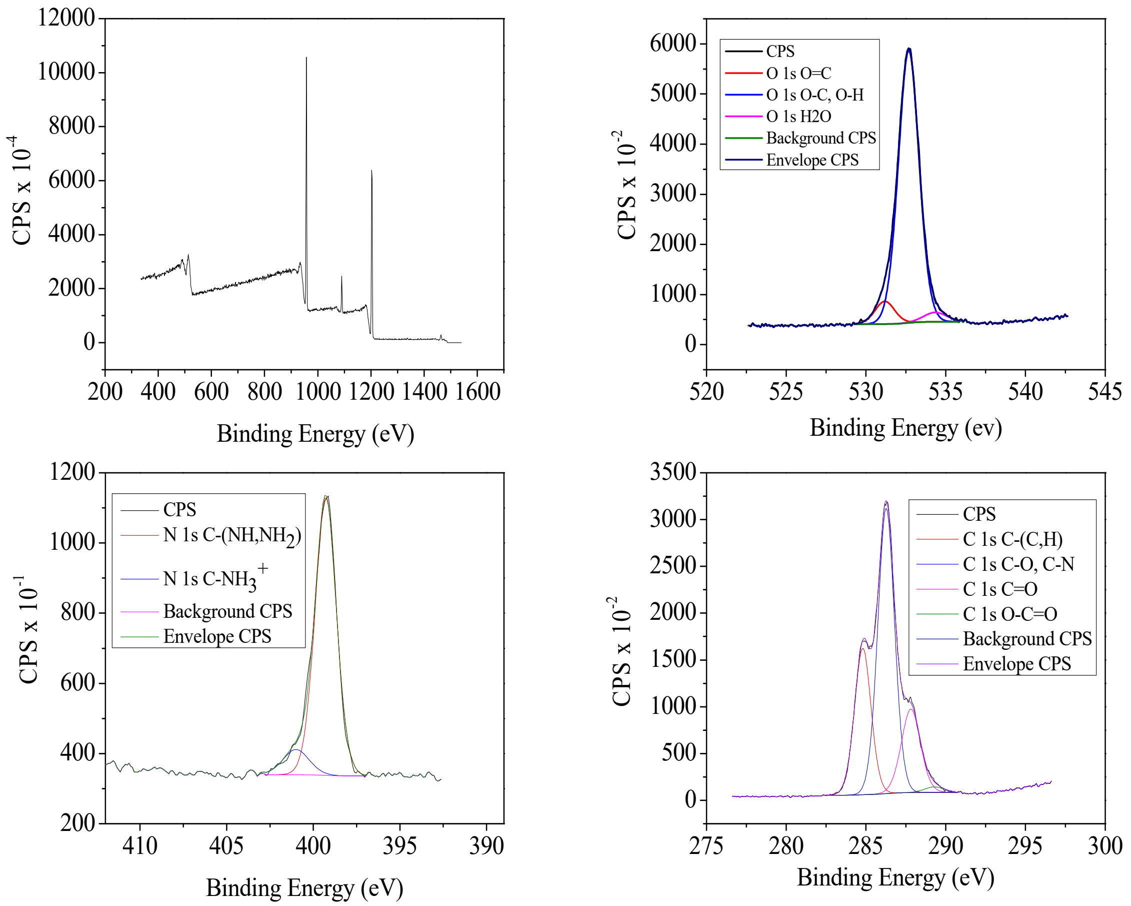

Figure S4. Representative XPS peaks: chitosan extracted, with peak decomposition and component position or assignment.

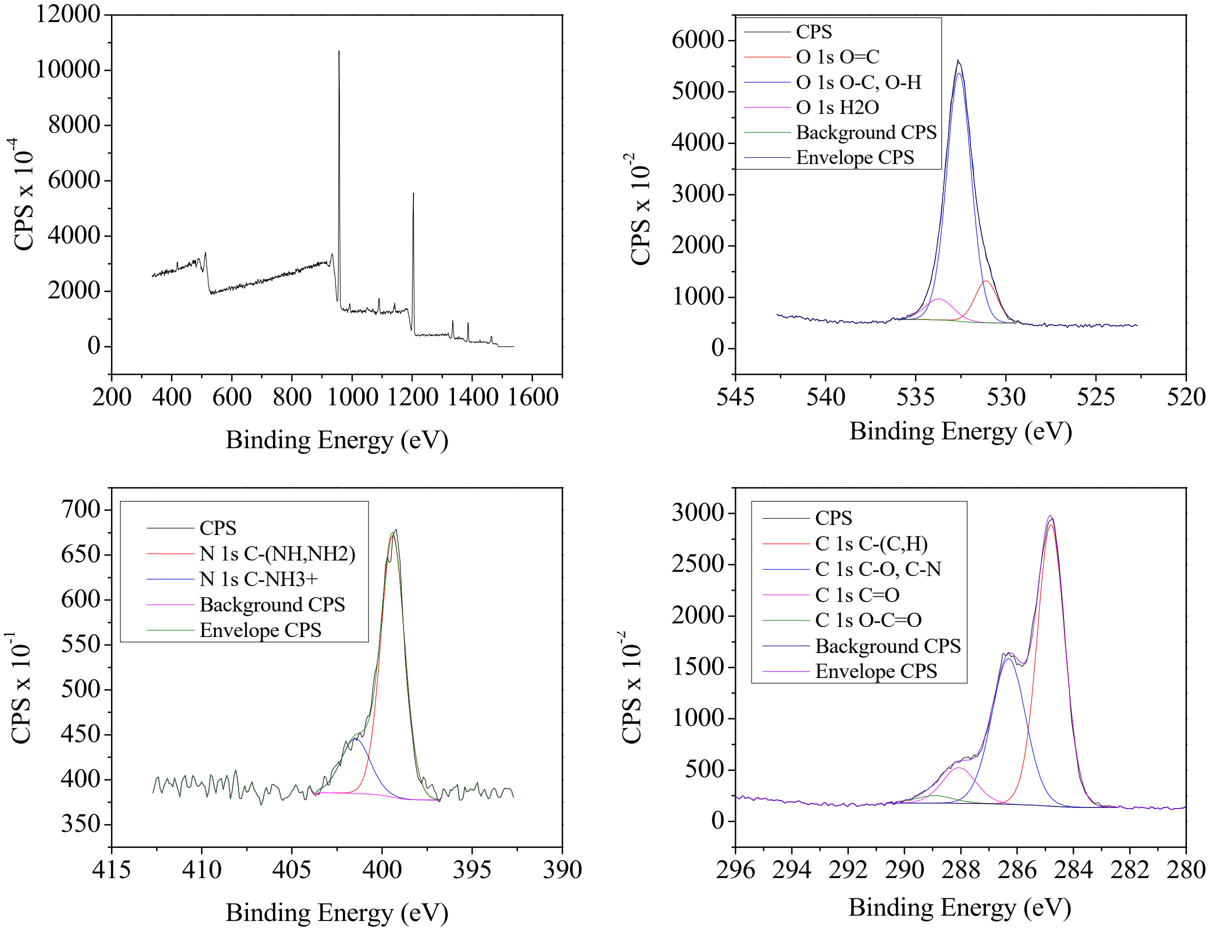

Figure S5. Representative XPS peaks: chitosan commercial, with peak decomposition and component position or assignment.

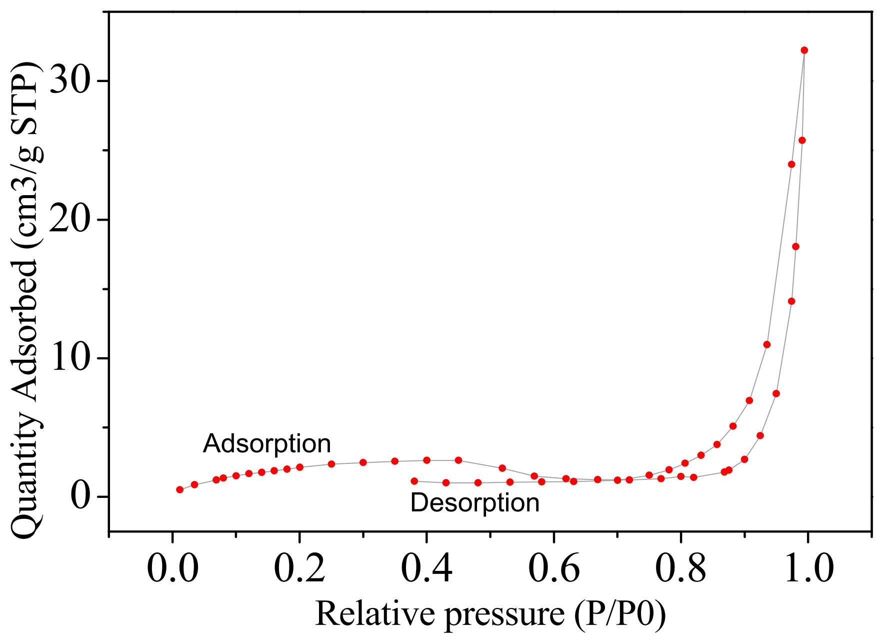

Figure S6. BET Isotherm Linear Plot of extracted chitosan.

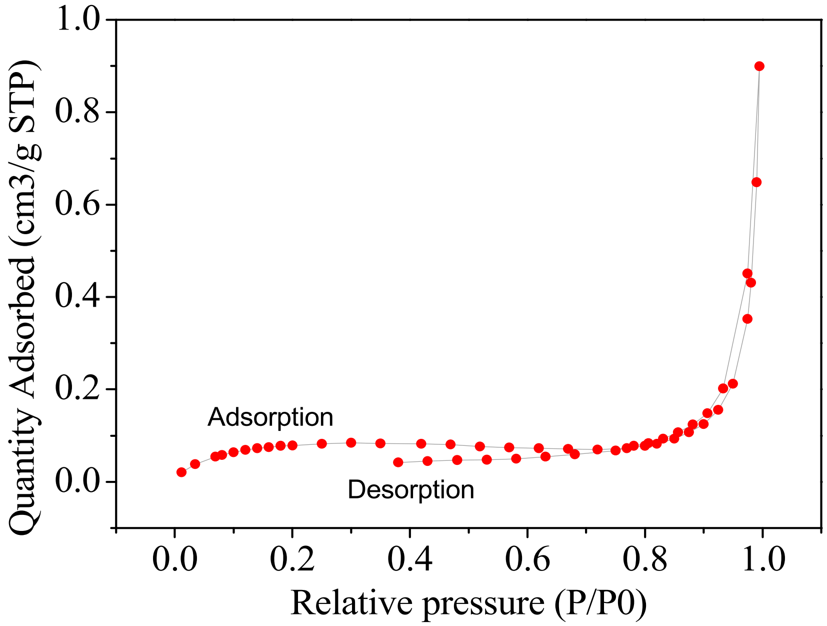

Figure S7. BET isotherm linear plot of commercial chitosan.

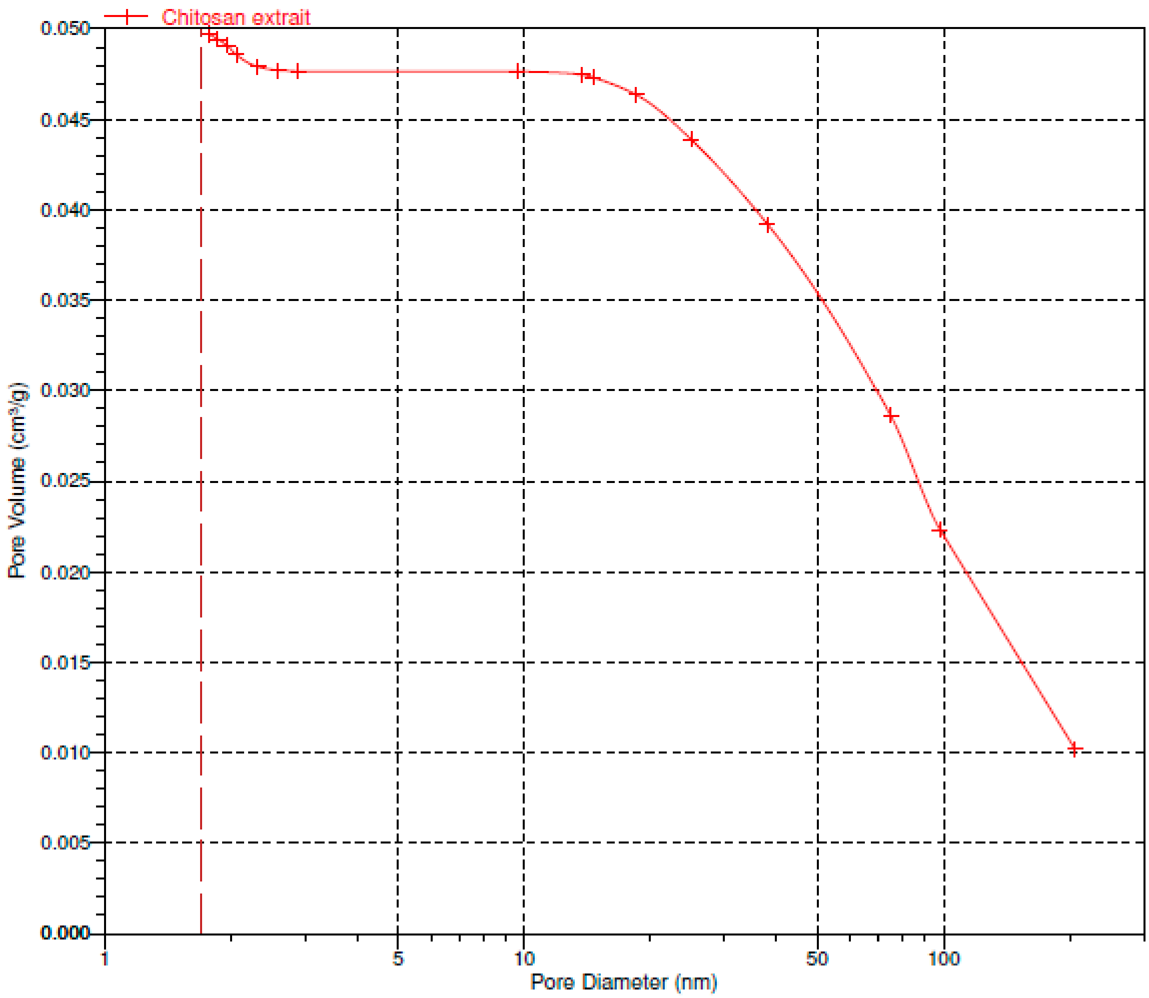

Figure S8. Pore volume in function of pore diameter of extracted chitosan.

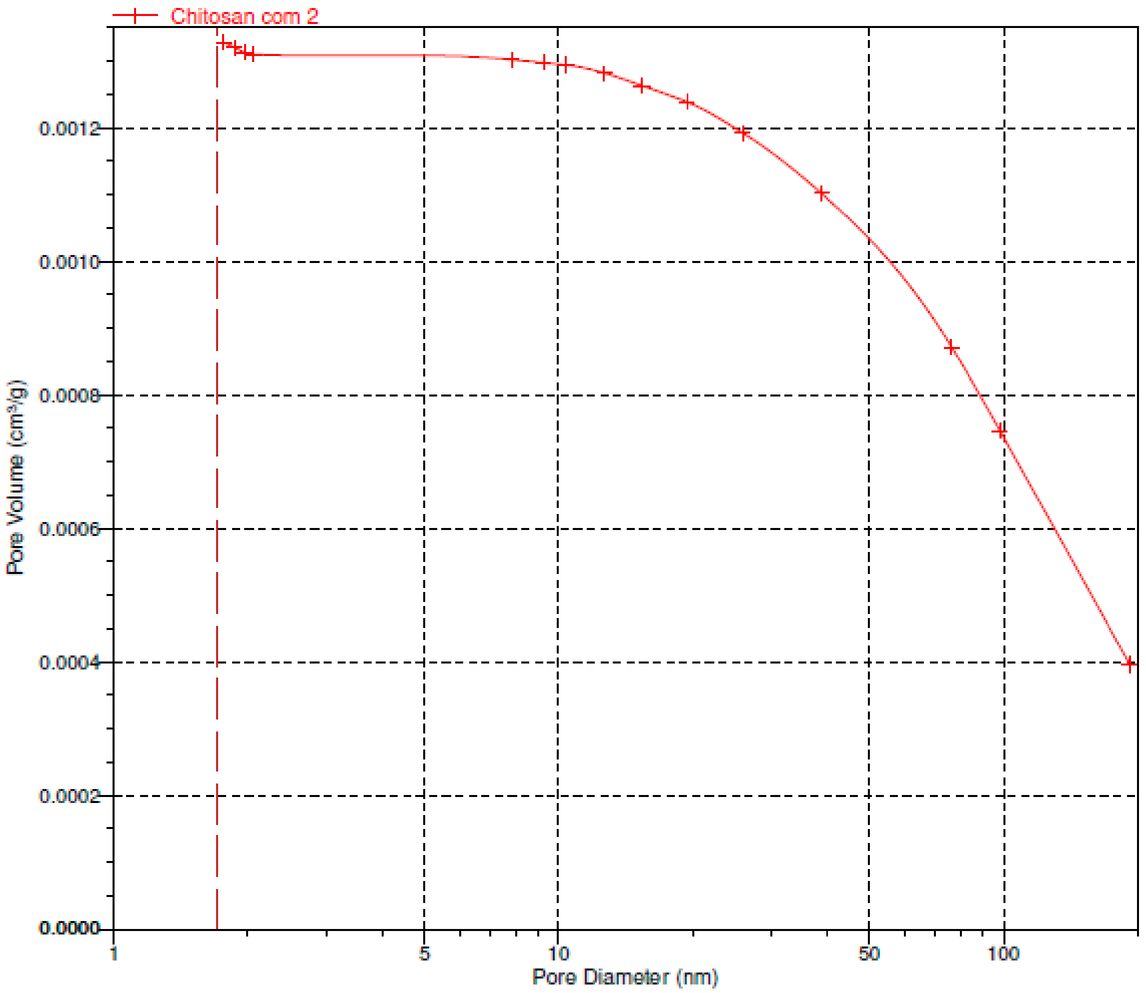

Figure S9. Pore volume in function of pore diameter of commercial chitosan.

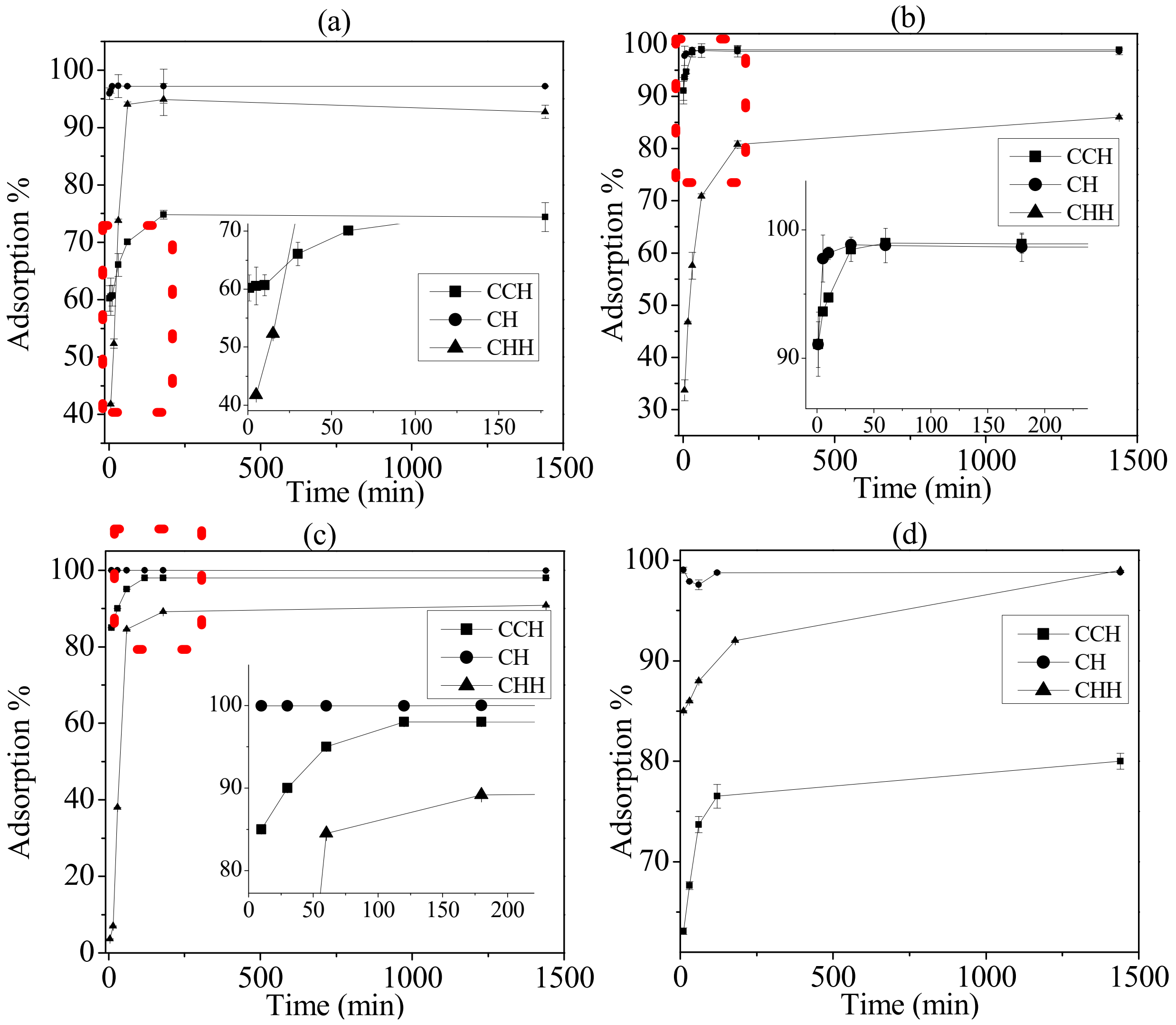

Figure S10. Adsorption of metals and dyes in function of time of contact, Pb ads (a), Cu ads (b), MB ads (c), CV ads (d).

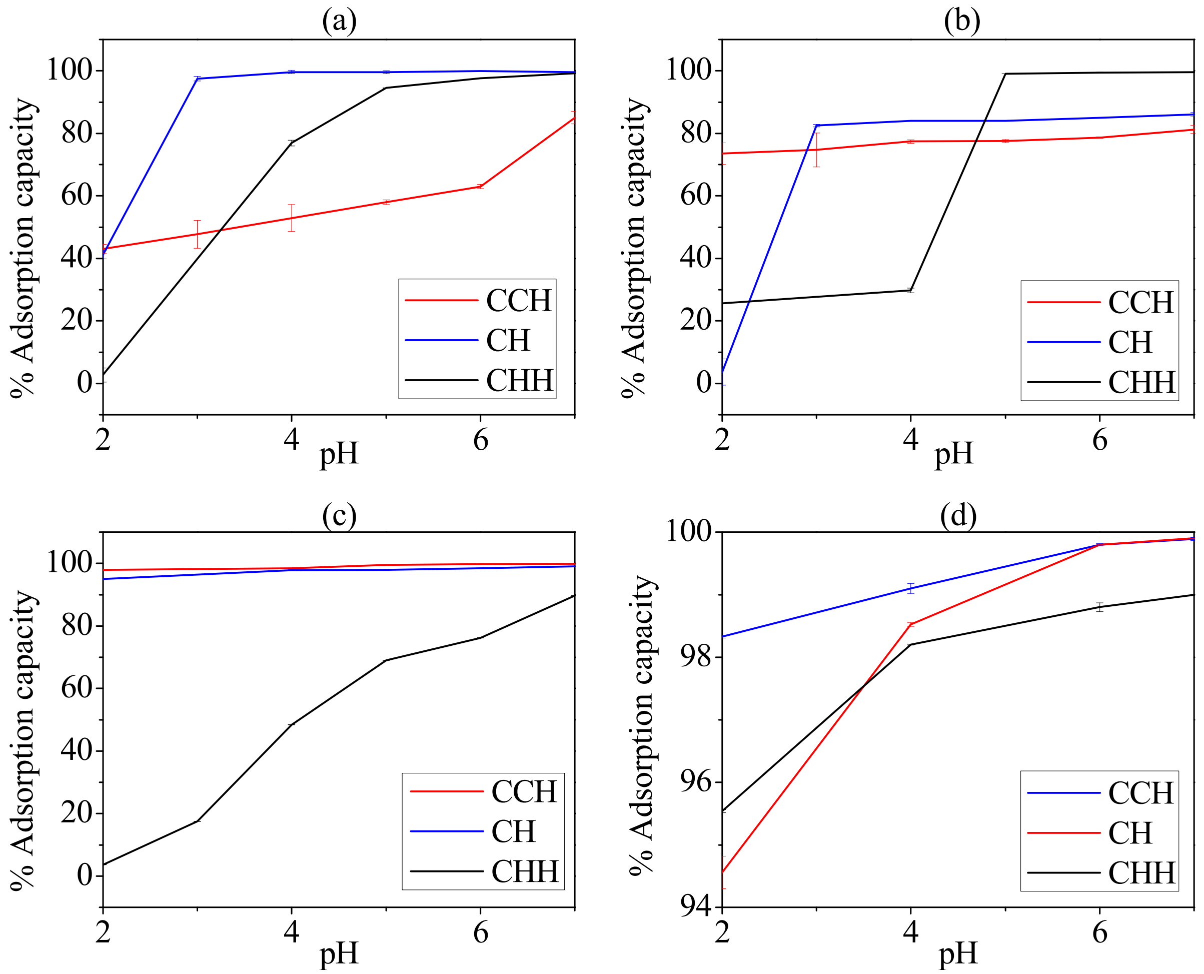

Figure S11. Adsorption of heavy metals and dyes in function of pH, Adsorption of lead in f(pH) (a), Adsorption of copper in f(pH) (b), Adsorption of MB in f(pH) (c), Adsorption f CV in f(pH)(d) with the three sample commercial chitosan (CCH), extracted chitosan (CH) and hydrogel chitosan (CHH).

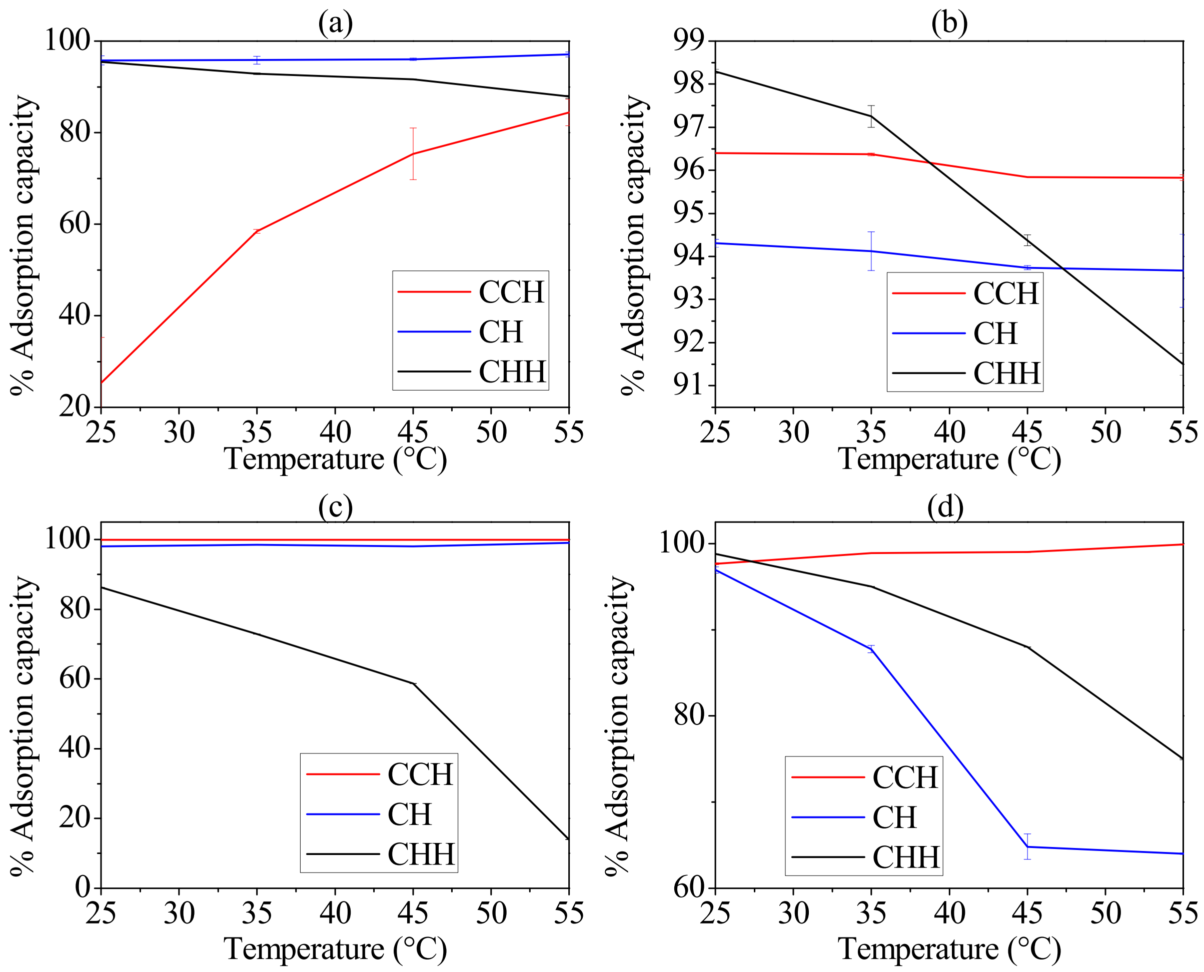

Figure S12. Adsorption of heavy metals and dyes in function of temperature, Adsorption of lead in f(temp) (a), Adsorption of copper in f(temp) (b), Adsorption of MB in f(temp) (c), Adsorption f CV in f(temp)(d) with the three sample commercial chitosan (CCH), extracted chitosan (CH) and hydrogel chitosan (CHH).

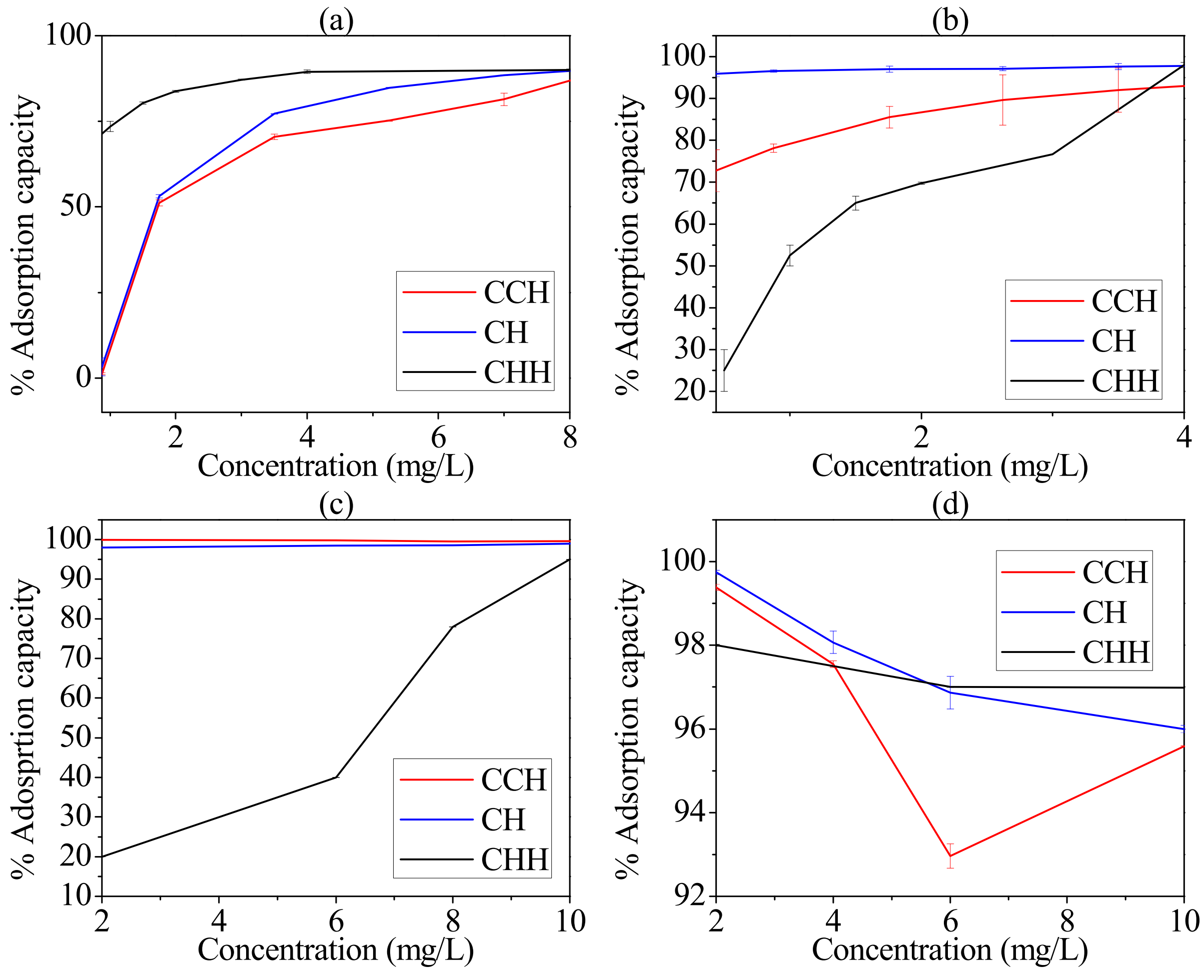

Figure S13. Adsorption of heavy metals and dyes in function of concentration, Adsorption of lead in f(conc) (a), Adsorption of copper in f(conc) (b), Adsorption of MB in f(conc) (c), Adsorption f CV in f(conc)(d) with the three sample commercial chitosan (CCH), extracted chitosan (CH) and hydrogel chitosan (CHH).

Adsorption isotherm models

Langmuir isotherm model: Under the assumptions of a uniform adsorbent surface, monolayer formation, and no interactions between the adsorbed molecules [64], the Langmuir linear equation can be expressed as follows:

$$\frac{Ce}{Qe} = \frac{1}{QmKL} + \frac{Ce}{Qm} \qquad \text{Eq (S1)}$$

In this equation, C_e represents the concentration of ions at equilibrium (mg L^{-1}), Q_e represents the adsorption capacity at equilibrium (mg g^{-1}), Q_m represents the maximum estimated adsorption at a monolayer (mg g^{-1}), and K_L represents the Langmuir constant associated with the sorption energy (L g^{-1}) [65].

Overview, the separation factor constant R_L, derived from the Langmuir equation, is calculated based on the following equation [66]:

$$RL = \frac{1}{1+KL\,C0} \qquad \text{Eq (S2)}$$

that C_0 is the initial metal ion concentration (mg L^{-1}).

Freundlich isotherm model: assumes the formation of multilayers on a heterogeneous surface during the adsorption process. The model is described by the Freundlich linear equation [68]:

$$\log Qe = \log KF + \frac{1}{nF} \log Ce \qquad \text{Eq (S3)}$$

In the Freundlich isotherm model, the adsorption capacity at equilibrium is denoted as Q_e (mg g^{-1}), the equilibrium concentration of ions is represented by C_e (mg L^{-1}), the Freundlich constant related to the adsorption capacity is denoted as K_F, and the Freundlich exponent indicating the adsorption intensity is represented as n_F [66,69].

Dubinin-Radushkevich isotherm model: describe the adsorption process on heterogeneous surfaces. It is based on the assumption that the adsorbent surface has a non-uniform pore size distribution. The Dubinin-Radushkevich equation is given by eqn (7):

$$Q = Qm\, e^{-\beta\, \varepsilon^2} \qquad \text{Eq (S4)}$$

Where in the Dubinin isotherm model, the adsorption capacity is denoted as Q (mol g^{-1}), the monolayer capacity of adsorbent as Q_m (mol g^{-1}), the Dubinin-Radushkevich constant related to the mean adsorption energy β (mol^2 kJ^{-2}), the Polanyi potential ε, which is calculated as

$$\varepsilon = RT \ln(1 + \frac{1}{C}) \qquad \text{Eq (S5)}$$

In this equation, R is the ideal gas constant (8.314 J mol^{-1} K^{-1}), T is the temperature in Kelvin, and C is the equilibrium concentration of the adsorbate (mol L^{-1})[71].

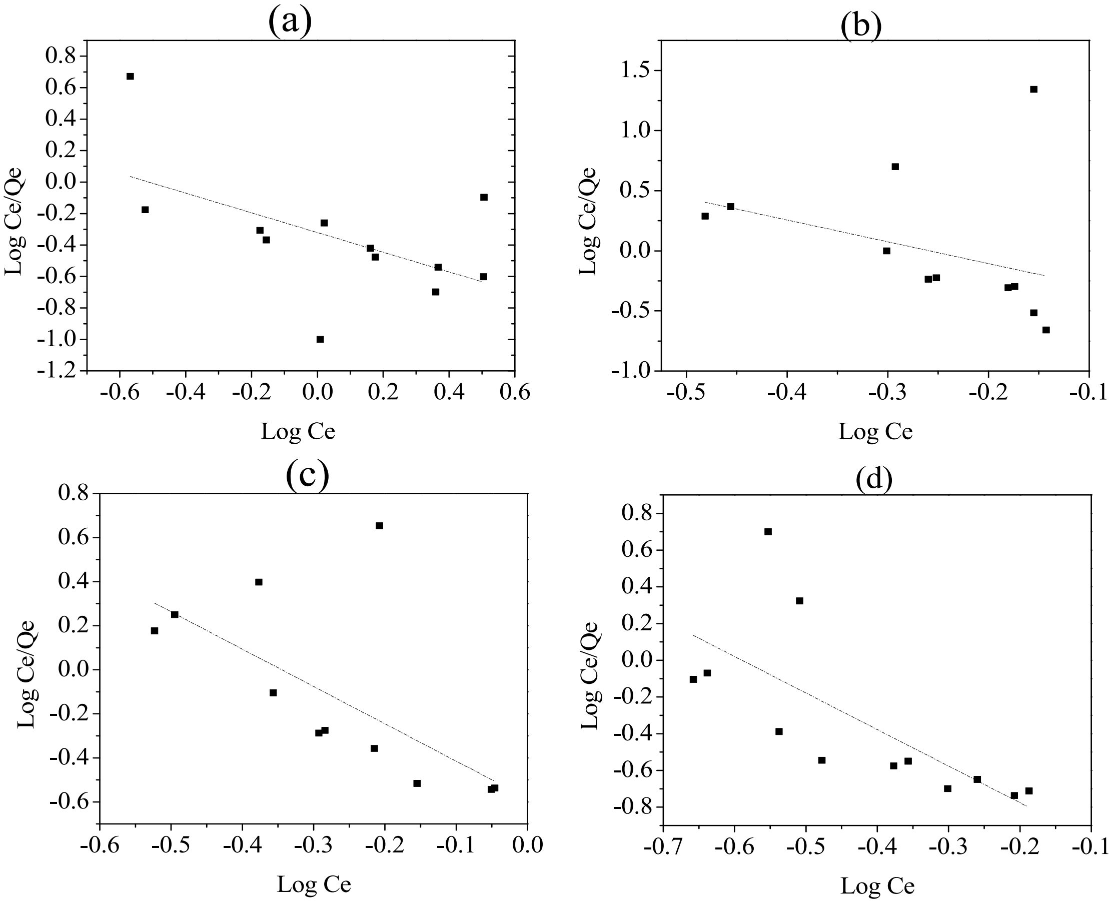

Figure S1. Isotherm Langmuir study for metals and dyes adsorption, copper for commercial chitosan (a), copper for extracted chitosan (b), lead for commercial chitosan (c), lead for extracted chitosan (d).

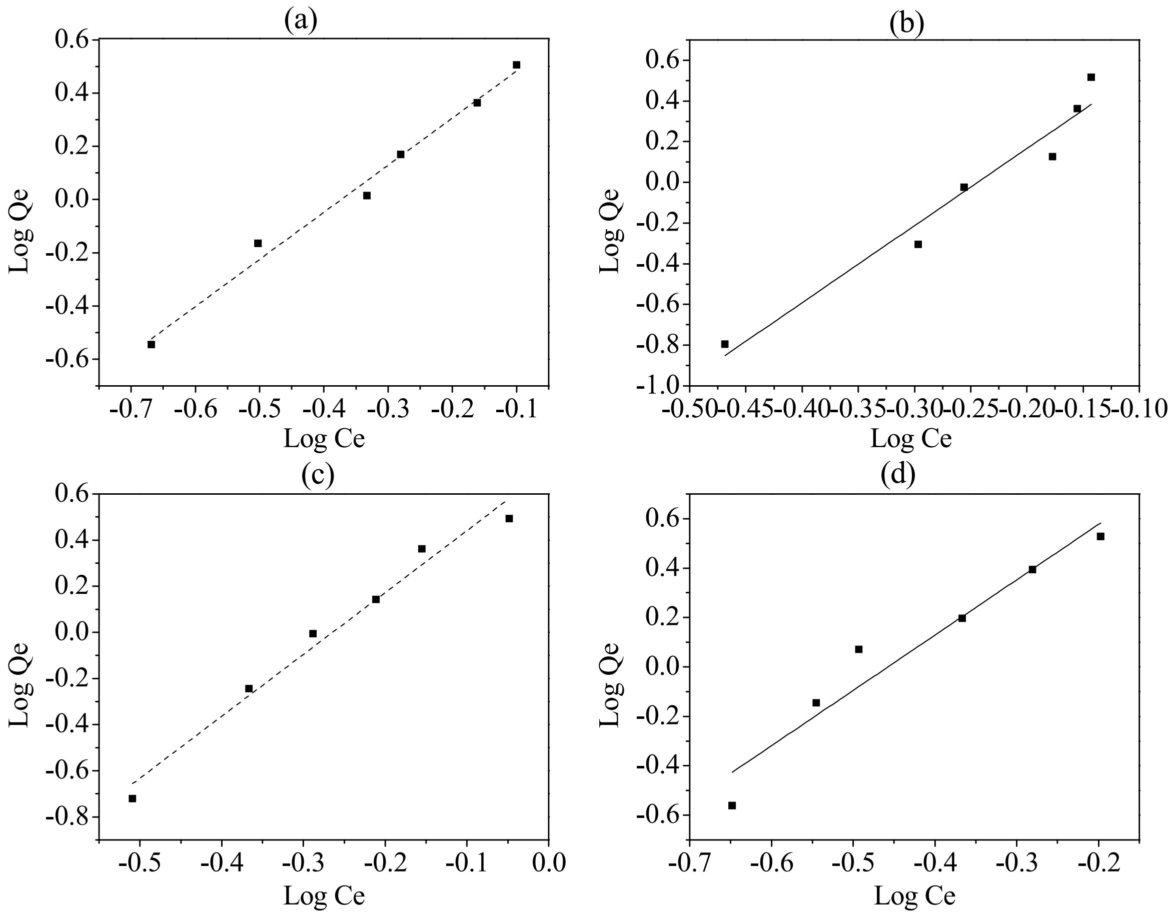

Figure S15. Isotherm Freundlich study for metals and dyes adsorption, copper for commercial chitosan (a), copper for extracted chitosan (b), lead for commercial chitosan (c), lead for extracted chitosan (d).

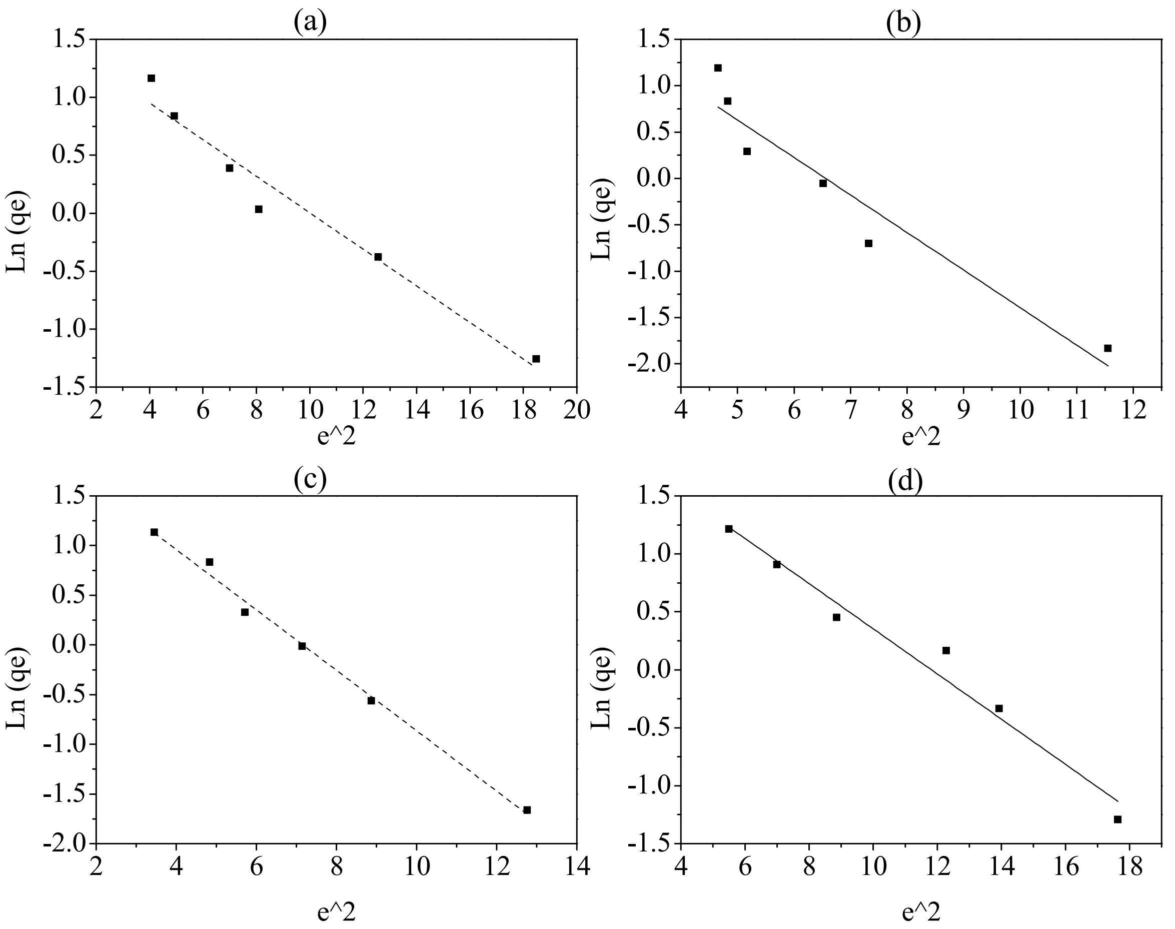

Figure S16. Isotherm Dubinin-Radushkevitch study for metals and dyes adsorption, copper for commercial chitosan (a), copper for extracted chitosan (b), lead for commercial chitosan (c), lead for extracted chitosan (d).

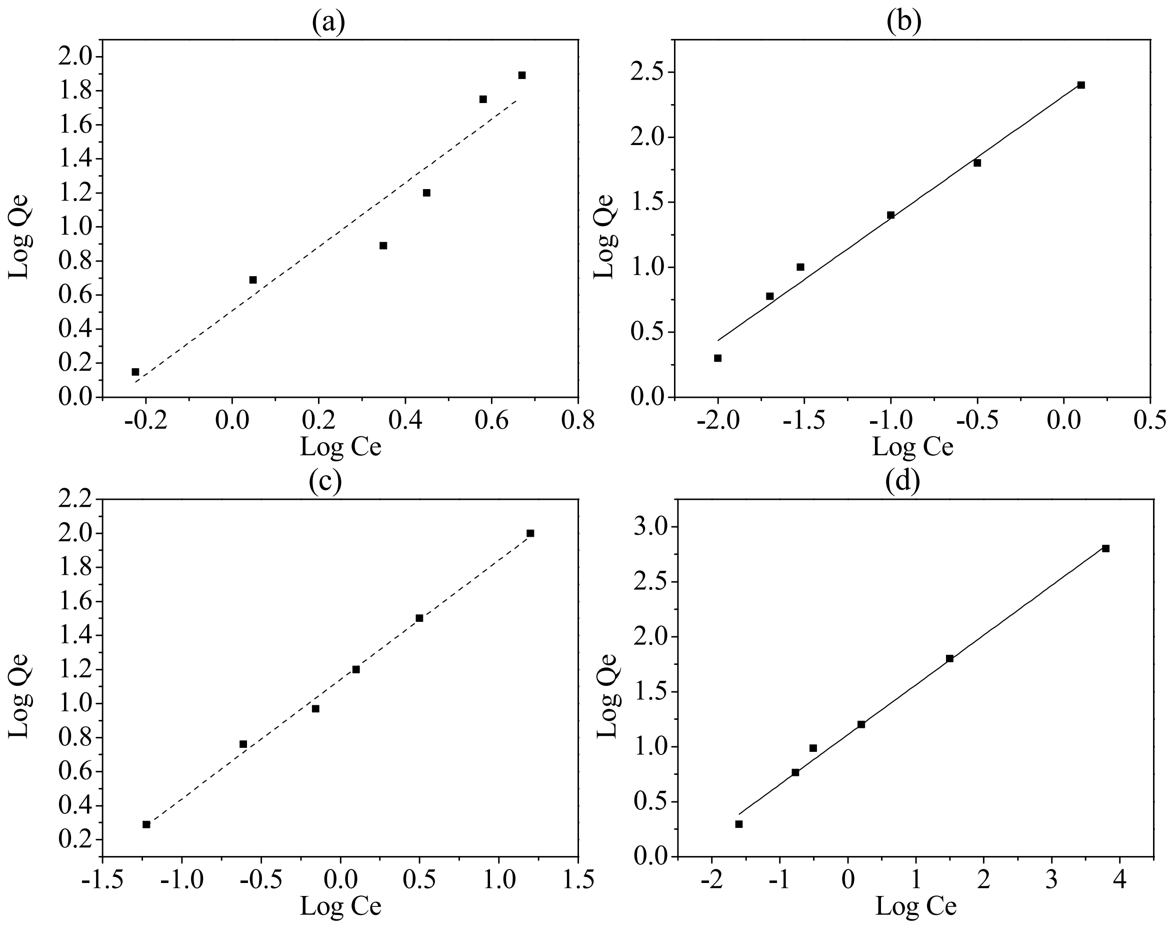

Figure S17. Isotherm Freundlich study for dyes adsorption, MB for commercial chitosan (a), MB for extracted chitosan (b), CV for commercial chitosan (c), CV for extracted chitosan (d).

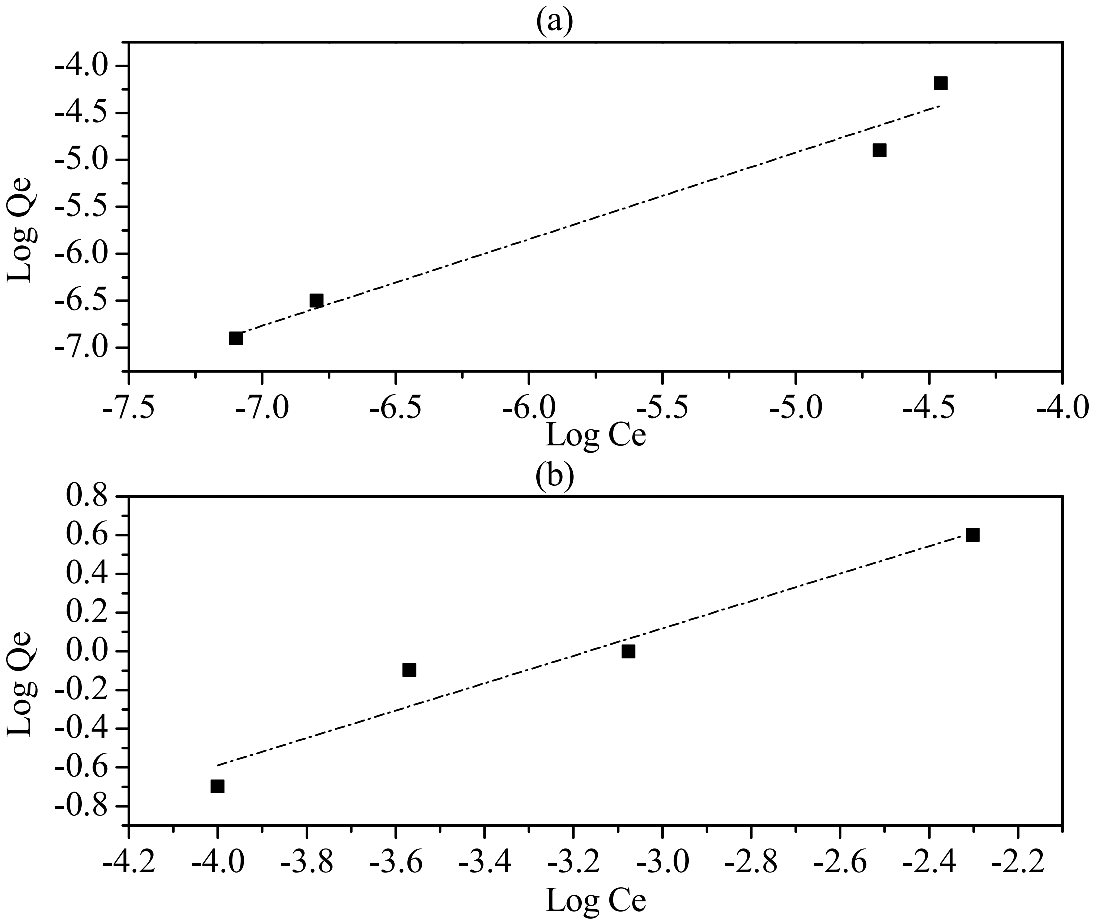

Figure S18. Freundlich isotherm study for dyes adsorption by chitosan hydrogel, for MB (a) and for CV (b).
